# Berlin Letter

**Published:** 1895-10

**Authors:** Julius Ullman


### Correspondence.

### BERLIN LETTER.

*Impressions of Professor Martin's Gynecological Clinic.*

DOCTOR A. MARTIN, whose father, the celebrated Edward Martin, was for a long period, and to his death, the professor ordinarius of obstetrics and gynecology at the University of Berlin, is a man of between forty-five and fifty years of age, six feet in height, very corpulent, weighing in the neighborhood of 300 pounds. He has a high forehead, full beard, a stern expression and is of domineering manner. Besides his native tongue, he speaks English, French and Italian fluently. He has the title of professor extraordinarius of obstetrics and gynecology at the University of Berlin, and during the year gives numerous post-graduate courses in diagnostic and operative gynecology.

During the course, all the students taking it are privileged and expected to attend all the operations at his private hospital. This is a very large, substantial brick structure, located in the central portion of the city. There are forty beds, divided into first, second and third class.

Professor Martin derives very much of his operative material from the "Poliklinik," which is situated in the lower portion of the building.

### LAPARATOMIES.

The operating room in which abdominal sections are performed is very small. Previous to the operation in the evening and early morning, it is thoroughly cleaned and the walls are saturated with a continuous carbolic acid spray.

Prof. Martin and his assistants, during the abdominal work, wear duck trousers, light shirts and rubber boots. Precisely at eight o'clock in the morning the patient, previously prepared, is brought stripped into the room and placed upon the operating table. This table, as well as many instruments used by Martin, was devised by Frau E. Horn, who has charge of the hospital and assists him in all his work.

The patient is placed in the lithotomy position. The table is low and short. Martin, wearing a huge rubber apron, sits at its lower end, the legs of the patient resting in his lap so that he is seated between them. Dr. Orthman, the first assistant, sits at the right of Martin; Frau Horn, having charge of the instruments, ligatures and sponges, stands at Martin's left.

Chloroform, made by Salamon & Co., England, is always used. There is continually at hand and ready for use an oxygen inhalation apparatus, and in one case it was to my knowledge used to great advantage.

The incision varies as the case requires. No hemostatic is used, for the parts are compressed digitally by Dr. Orthman, who also forces the intestines from the field of operation. For hemorrhage into the peritoneal cavity aseptic sponges are used. All ligatures are tied with catgut. The stumps are never treated with the Paquelen cautery.

Before closing the wound, a sterilised sponge, saturated with fresh neutral olive oil, is placed into the peritoneal cavity so as to prevent the formation of adhesions, and prior to suturing the abdominal wall the sponge is removed and the excess of oil, as well as air and any remaining clots or coagula, are expelled by manual compression. The abdominal wall and peritoneum are sutured with two to three stay sutures of heavy braided silk and intermediate catgut sutures.

If in any of his operations Martin finds pus he does not use drainage of any kind, claiming that anything, be it a tube or gauze used for this purpose, acts as a foreign body to the tissues, and, because of the irritation resulting, produces a secretion. He has good results by his method, which is, after thorough cleansing of a pus cavity, closing the wound as any other, and depending on phagocytosis for absorption.

The Trendelenberg position is never used, for, as previously noted, Martin always sits during the laparatomies.

The dressing of the wound is very simple; sterilised absorbent cotton, kept in place by strips of adhesive plaster, over which there is wound a roller gauze bandage.

OPERATIONS OTHER THAN ABDOMINAL SECTIONS.

The plastic operations and vaginal hysterectomies are performed in another operating-room. The patient is placed in the lithotomy position, and the Simons speculum is used exclusively; an assistant on either side holding a leg and a retractor.

Emmet's trachelorrhaphy and Martin's perineorrhaphy are performed frequently. If there is a small ovarian cyst, hematoma or sacto salpinx serosa, hemorrhagica or purulenta, without too many adhesions, he removes them by a corporotomy. For retroflections or retroversions requiring operative interference, Professor Martin performs, instead of the Alexander operation or ventral hysterorrhaphy, a vaginal fixation, by which the corpus uteri is sutured to the anterior vaginal wall. This operation was described with excellent results by both Mackenrodt and Dührssen, of Berlin, and is now frequently used at the various gynecological clinics in Berlin and Vienna.

Again, in all these operations, catgut only is used. For a dressing, in these cases, small strips of iodoform gauze are placed into the vagina. No outer dressing is used; the thighs are tied.

Of interest in the preparation of the vagina before operation is the fact that the vagina is not only washed with soap and water, then irrigated with 1-2000 mercuric chlorid, and absolute alcohol, but that it is packed on the night previous to the day of operation with 1-2000 mercuric chlorid gauze, this packing being renewed in the morning two hours before operation.

Professor Martin prefers, whenever he can do it, vaginal hysterectomy to the abdominal method, for he cannot overcome the great danger of ventral hernia which laparatomies have in general.

Interesting from a bacteriological point of view is the preparation of the culture medium used at the Martin Hospital. Through the courtesy of Doctor Kiever, one of the assistants, I am allowed to present the method. Ascitic fluid, be it from hepatic cirrhosis, tubercular or carcinomatous peritonitis, is used. The ascitic fluid from carcinomatous peritonitis is the most advantageous, for it contains the greatest amount of albumin.

The fluid is taken unfiltered and sterilised for six to eight days by fractional sterilisation at a temperature of 63° C. An equal amount of sterilised agar agar is then added to it. The culture medium is poured into Petri vessels and is ready for use.

Through the kindness of Frau E. Horn I am enabled to give the method of preparation of catgut, which is used with such excellent results at the hospital.

The catgut is made by Marke-Weimar and is to be had from Boehme, in Berlin. It is wound on glass plates and then placed for six hours (no longer, else it becomes brittle and breaks,) in a one to one thousand (1–1000) mercuric chloride solution. It is then placed into a solution of one part juniper oil and two parts absolute alcohol, in which it remains twelve hours. This dehydrates and removes the fat from the catgut. Another similar solution—one part juniper oil and two parts absolute alcohol—is made and the catgut placed into it, remaining for at least fourteen days before use. It should be kept immersed in a glass-stoppered jar. In this process the catgut is not boiled and therefore does not soften.

Too much praise cannot be bestowed on Frau E. Horn. She is an admirable assistant, is very alert and knows every step of all operations, so that Professor Martin is never compelled to speak or beckon for anything during his operative work. Having from her childhood been reared in environments where she saw the nursing of the sick and wounded, it remained for the breaking out of hostilities of the Franco-Prussian war to decide her, then in her eighteenth year, to undertake her life-work. She served as volunteer nurse throughout the war, and at its close entered the "Frauen klinik" under Professor Schrödter. She soon left this position and for the past seventeen years has associated herself as assistant to Professor Martin. She is in the neighborhood of forty-five years of age, is plump, has a merry face, a twinkling eye, an all-round comfortable manner, and is always ready for a smile. She does her work not out of necessity, but wholly because of the satisfaction, pleasure and joy which it gives her. She is a very practical woman and, as previously stated, has devised the operating table sold under her name, many instruments and the method of preparation of catgut, decribed above. She is a woman of great wealth and possesses a castle at Eberswalde, which is a beautiful town in the "Marksche Schweiz," about one hour's railway ride from Berlin.

On a Saturday evening, when finished with her week's work, she seeks the quiet and solitude of her charming estate. It is then, as she says, "I live," but early on the following Monday morning she is busy at her accustomed work again.

JULIUS ULLMAN, M. D.

